# Prevalence of and risk factors associated with *Cryptosporidium* infection in an underdeveloped rural community of southwest China

**DOI:** 10.1186/s40249-016-0223-9

**Published:** 2017-01-09

**Authors:** Ya Yang, Yi-Biao Zhou, Peng-Lei Xiao, Yan Shi, Yue Chen, Song Liang, Wu-Li Yihuo, Xiu-Xia Song, Qing-Wu Jiang

**Affiliations:** 1 School of Public Health, Fudan University, Building 8, 130 Dong’an Road, Xuhui District, Shanghai, 200032 China; 2 Key Laboratory of Public Health Safety, Fudan University, Ministry of Education, Building 8, 130 Dong’an Road, Xuhui District, Shanghai, 200032 China; 3 Center for Tropical Disease Research, Fudan University, Building 8, 130 Dong’an Road, Xuhui District, Shanghai, 200032 China; 4 School of Epidemiology, Public Health and Preventive Medicine, Faculty of Medicine, University of Ottawa, 451 Smyth Road, Ottawa, ON K1H 8M5 Canada; 5 Department of Environmental and Global Health, College of Public Health and Health Professions, University of Florida, 2055 Mowry Road, Gainesville, FL 32611 USA; 6 Emerging Pathogens Institute, University of Florida, 2055 Mowry Road, Gainesville, FL 32611 USA; 7 Puge Center for Disease Prevention and Control, 6 Qingnian Road, Puge County, Sichuan, 615300 China

**Keywords:** *Cryptosporidium*, Hepatitis B virus, Human immunodeficiency virus, Prevalence, Risk factors, Rural areas, China

## Abstract

**Background:**

*Cryptosporidium spp.* is an important intestinal protozoan causing diarrhea in humans, livestock, and wild animals. *Cryptosporidium* infection remains a major public health issue, but its epidemiology in humans is still unclear, particularly in rural China. This study was designed to determine the prevalence of and risk factors associated with *Cryptosporidium* infection in a rural southwestern Chinese community.

**Methods:**

A community-based cross-sectional survey was conducted among 687 residents of a small town in a Yi autonomous prefecture of southwest China in 2014. Blood samples were examined using a broad set of quality-controlled diagnostic methods for hepatitis B virus (HBV) and human immunodeficiency virus (HIV). Stool specimens were processed using the modified acid-fast staining method, and microscopically examined for *Cryptosporidium* infection. Univariable and multivariable analyses were performed to determine the risk factors associated with *Cryptosporidium* infection.

**Results:**

The majority of the participants were Yi people with poor living conditions and unsatisfactory hygiene habits, and the study area was of very low socioeconomic status. Of the 615 individuals included in the analysis, 14 (2.3%) were HIV positive, 51 (8.3%) were infected with HBV, and 74 (12.0%) had *Cryptosporidium* infection. The prevalences of HIV/HBV, HIV/*Cryptosporidium*, and HBV/*Cryptosporidium* co-infections were 0.3%, 0.3%, and 1.8%, respectively. The prevalence of HBV infection was higher in individuals with *Cryptosporidium* infection (*χ* ^*2*^ *=* 5.00, *P* = 0.03). Owning livestock or poultry was an important risk factor for *Cryptosporidium* infection (a*OR* = 2.27, 95% *CI*: 1.01–5.08, *P* < 0.05). *Cryptosporidium* infection was significantly associated with HBV infection (a*OR* = 3.42, 95% *CI*: 1.47–7.92, *P* < 0.01), but not with HIV infection (a*OR* = 0.57, 95% *CI*: 0.07–4.39, *P* = 0.59).

**Conclusions:**

The prevalence of *Cryptosporidium* infection was high in the rural area of southwestern China that was investigated, and there was a significant association between HBV infection and *Cryptosporidium* infection. Further investigations are needed to determine the significance of *Cryptosporidium* infection in patients infected with HBV.

**Electronic supplementary material:**

The online version of this article (doi:10.1186/s40249-016-0223-9) contains supplementary material, which is available to authorized users.

## Multilingual abstracts

Please see Additional file 1 for translations of the abstract into the five official working languages of the United Nations.

## Background

*Cryptosporidium* is an important protozoan parasite, which causes diarrheal disease in humans worldwide [1]. The Global Enteric Multicenter Study assessed the causes, burden, clinical syndromes, and adverse outcomes of moderate-to-severe diarrhea involving more than 20 000 children in Sub-Saharan Africa and South Asia, and found that *Cryptosporidium* was second only to rotavirus as a cause of moderate-to-severe diarrhea in children younger than 2 years [2]. Although *Cryptosporidium* was first discovered in 1907, it was not until 1976 that this parasite was identified as a cause of human infection [3]. In 2004, cryptosporidiosis was added to the World Health Organization’s ‘Neglected Diseases Initiative’ , which includes diseases affecting people mainly in low-resource settings [4].

In immunocompetent individuals, *Cryptosporidium* infection may be asymptomatic or cause self-limiting diarrhea. In immunocompromised patients such as those with human immunodeficiency virus (HIV)/acquired immune deficiency syndrome (AIDS), however, *Cryptosporidium* may cause severe, chronic, and possibly life-threatening diarrhea, and profound malnutrition or wasting. Despite the relative consensus of opinion regarding the severity of *Cryptosporidium* infection in patients with HIV/AIDS, there does not seem to be a shared understanding of the risks for other groups of immunosuppressed individuals, including those with hepatitis B virus (HBV) infection. There is mounting evidence indicating that the immune system is compromised in patients with HBV [5]. However, data on the epidemiology of *Cryptosporidium* infection in individuals infected with HBV is scarce.

Numerous studies have demonstrated the critical importance of cryptosporidiosis, but *Cryptosporidium* failed to convince the pharmaceutical industry that a market for new therapeutics exists. Consequently, there is no specific therapeutic agent available. In the absence of effective specific treatment and a lack of access to health care, *Cryptosporidium* continues to be a major cause of opportunistic infections in developing countries.

In China, the first human cases of cryptosporidiosis were reported in 1987 in Nanjing, Jiangsu Province [6]. Since then, increasing attention has been paid to *Cryptosporidium*, and a number of epidemiological investigations have confirmed the existence of human cryptosporidiosis in at least 14 out of the 32 provinces in China [7]. However, previous studies have focused on children or patients with diarrhea or HIV/AIDS [7, 8], and few studies were designed to investigate the overall prevalence and epidemiology of cryptosporidiosis of the general population in rural areas, and none have been conducted among people other than the Han nationality [7, 9].

We conducted a cross-sectional study in an underdeveloped region inhabited by the Yi people, an ethnic minority group in P.R. China, with a high prevalence of HIV/AIDS and HBV [10, 11]. Local farmers in this community are engaged in mixed agricultural practices and use animal manure and human excreta as important organic fertilizers. People have close and frequent contact with domestic animals, and there is a lack of access to sanitation facilities. The primary aim of this research was to determine the prevalence of *Cryptosporidium* infection, and its association with HIV and HBV and other risk factors in the region.

## Methods

### Study area and participants

This community-based cross-sectional study was carried out in M town, P County of a Yi autonomous prefecture, southwest China. This town covers about 50 km^2^, with a population of approximately 4 000, and has a complex topography with mountains and valleys at an elevation of 1 800 to 2 500 m [12]. The main rainy season is from May to October, although occasional rain occurs at all times of the year. According to meteorological statistics, the average temperature for the region is about 16.8 °C and the average rainfall is about 1 170 mm per year. Winters feature mild days and cool nights, whilst summers are very warm and humid.

The residents are engaged in husbandry, and produce food crops such as corn, potato, oats, and vegetables. Domestic animals outnumber humans in the area. Agriculture and animal husbandry employ more than 90% of the population. It was not until 2006 that lavatories, latrines, or any other forms of sanitation facilities became available. Until then, both human and domestic animal feces were left undisposed [12].

**Fig. 1:**
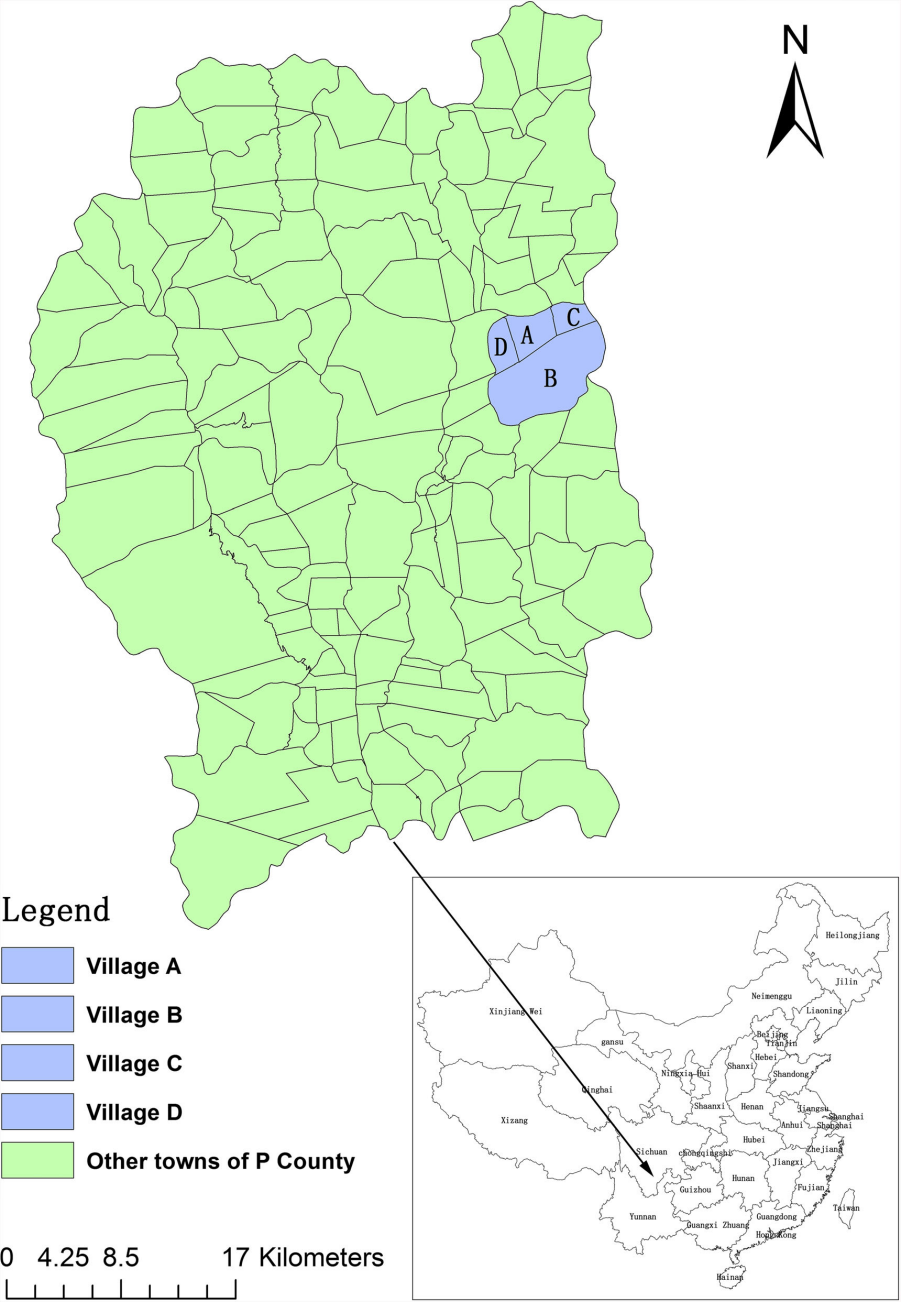
Administrative map of study area (P County of a Yi autonomous prefecture, southwest China). M town consists of four villages named A, B, C, and D

Participants who had lived in the area for over 6 months were invited to participate in the study. Participants who had severe organic or mental diseases and pregnant women were excluded. A total of 687 individuals were randomly selected from four villages (named A, B, C, and D, as shown in Fig. 1).

### Study procedures

The study was carried out from 23rd October until 3rd November 2014. After a brief introduction of the study at the site, each of the participants from the study villages was registered and given a clean plastic container for collecting a single stool sample of at least 30 g in the morning at home. Specific instructions were given for proper collection and avoidance of possible contamination. A structured questionnaire was administered to each participant by fieldworkers from the local Center for Disease Control and Prevention (CDC) to gather information about sociodemographic characteristics (including name, age, gender, ethnicity, marital status, education, occupation, annual family income and keeping livestock or poultry), personal hygiene habits (including drinkng unboiled water, washing hands before meals and after defecation and washing before eating raw fruits and raw vegetables) and access to safe water and sanitation facilities.

All the participants had a finger prick to obtain about 1 ml of blood and were screened for HBV surface antigen and anti-HIV antibodies in the field using the diagnostic test kit for HBV (colloidal gold) (Livzon Pharmaceutical Group Inc., Zhuhai, P. R. China) and the diagnostic test kit for HIV (colloidal gold) (Xiamen Interactive Technology Co., Ltd., Xiamen, P. R. China). Positive individuals were asked to provide 5 ml blood specimen for conformation. These blood samples were stored in the CDC laboratory refrigerator, which was kept at a constant temperature of −20 °C. Conformation tests were conducted by using the diagnostic kits for quantification of HBV DNA and HIV-1 RNA (polymerase chain reaction fluorescence probe method) (DAAN Gene Group Inc., Zhongshan, P. R. China) at the Center for Tropical Disease Research, Fudan University.

Stool samples were collected once for each participant and sent to the laboratory of the local CDC as soon as possible. All the specimens were processed on the day of collection, using modified acid-fast staining. Thin smears were prepared from freshly collected stool samples, and were air dried before fixation (5 s on a candle flame). The smears were stained with carbol fuchsin for 10 min and thereafter washed with tap water. The slides were decolorized with 3% acid alcohol for 2 min and were counterstained with methylene blue for another 30 s and dried at room temperature. Finally, the stained smears were examined using immersion oil to detect oocysts of *Cryptosporidium*. In this method, *Cryptosporidium* oocysts appear as pink to red, spherical to ovoid bodies on a blue background. A stool was labeled as positive if the size of the oocysts ranged between 4 μm and 6 μm.

### Quality control

Fieldworkers were specifically trained under the guidance of a unified protocol prior to the field investigation. Two slides were prepared for each sample, and were examined by two separate laboratory technicians from the Center for Tropical Disease Research, Fudan University. The laboratory staff was not informed about the health status of the study participants to minimize observer bias. A third senior technician was summoned if there was disagreement concerning the results. All smears were stored in boxes at room temperature, and 10% of the examined slides were randomly selected and re-examined for quality control. All data were checked for accuracy.

### Data analysis

Data were double entered and crosschecked with EpiData software (version 3.1; The EpiData Association, Odense, Denmark). Descriptive summary measures of frequency and central tendency of participants’ characteristics were computed as appropriate. Pearson’s chi-square test was employed in the univariable analysis to examine the associations between participants’ characteristics and infection with HBV, HIV, and *Cryptosporidium* by computing crude odds ratios (c*OR*s) with 95% confidence intervals (*CI*s). Multivariable logistic regression was subsequently carried out through stepwise elimination to adjust for confounders, and adjusted *OR*s (a*OR*s) with 95%*CI*s were calculated. *P* ≤ 0.05 were considered statistically significant. Statistical analyses were done with the SPSS statistical package (version 20.0; IBM SPSS Institute, Inc., USA).

### Ethics statement

The study was approved by the Ethics Review Committee of the Ethical Institute of the School of Public Health, Fudan University. Community leaders and local health authorities were informed during sensitization meetings about the purpose, procedures, potential risk, discomforts, and benefits of the study prior to the survey and sample collection.

All participants provided consent and were informed of their right to refuse to participate or withdraw at any point during the study. Written informed consent was obtained from the parents/guardians of children prior to study procedures after they had been clearly informed about the study. Participants infected with *Cryptosporidium* or with other medical conditions received appropriate treatment/referral according to the local treatment policies and national guidelines. Identity and information collected were kept confidential.

## Results

A total of 687 individuals were investigated in the study. Of them, 615 participants provided both blood and stool specimens, and were included in the analysis. There were no significant differences between participants included and those excluded in terms of gender, age, ethnicity, education, and annual family income.

The sociodemographic characteristics of the participants are summarized in Table 1. Participants were predominantly Yi people (99.8%), of whom 251 (40.8%) were male and more than half were married (62.3%). The majority of the participants were illiterate (62.1%) and farmers (80.0%). The median age of the study participants was 34 years with an interquartile range from 14 to 47 years. The majority of the residents (97.7%, 589/603) drank unboiled water, two-thirds (68.2%, 392/575) never or occasionally washed fruits or vegetables, almost half hardly or occasionally washed their hands before meals or after defecation, and 40.4% (239/591) had no access to a household lavatory.

**Table 1:** Characteristics of the study population

Variables	No. of participants	%
Total	615	100.0
Village		
A	172	28.0
B	158	25.7
C	129	21.0
D	156	25.4
Gender		
Male	251	40.8
Female	364	59.2
Age (years)		34 (14, 47)^a^
0–10	110	17.9
11–20	75	12.2
21–30	87	14.1
31–40	118	19.2
41–50	110	17.9
51–60	64	10.4
>60	51	8.3
Ethnicity		
Yi	614	99.8
Han	1	0.2
Marital status		
Not married	194	31.5
Married	383	62.3
Widowed	38	6.2
Body mass index (BMI, kg/m^2^)		20.4 (19.1,22.2)^a^
>18	526	85.5
≤18	89	14.5
Education		
Illiterate	382	62.1
Primary school	206	33.5
Middle school and above	27	4.4
Occupation		
Student	113	18.4
Farmer	492	80.0
Other	10	1.6
Annual family income (yuan)		
<3 000	117	19.0
3 000–5 000	188	30.6
5 000–10 000	116	18.9
>10 000	194	31.5
Keeping livestock or poultry (e.g., cattle, sheep, chicken)		
No	155	25.2
Yes	460	74.8
Drinking unboiled water		
No	14	2.3
Yes	589	97.7
Washing hands before meals		
Often or always	352	58.2
Never or occasionally	253	41.8
Washing hands after defecation		
Often or always	329	54.3
Never or occasionally	277	45.7
Washing fruits and raw vegetables before eating them		
Often or always	183	31.8
Never or occasionally	392	68.2
Having a household lavatory		
No	239	40.4
Yes	352	59.6
Living under the same roof with livestock		
No	541	93.9
Yes	35	6.1

^a^Values are median (interquartile range)

The prevalences of HIV, HBV, and *Cryptosporidium* were 2.30%, 8.30%, and 12.0%, respectively (see Table 2). The prevalences of HIV/HBV, HIV/*Cryptosporidium*, and HBV/*Cryptosporidium* co-infections were 0.3%, 0.3%, and 1.8%, respectively. Prevalence of *Cryptosporidium* was highest among individuals aged 41–50 years, followed by elderly adults aged ≥ 60 years and children aged 0–10 years (see Table 4). No study participants had a multiple infection involving HBV, HIV, and *Cryptosporidium*. Results in Table 3 show that the prevalence of HBV infection was higher in individuals with *Cryptosporidium* infection (*χ* ^*2*^ *=* 5.00, *P* = 0.03).

**Table 2:** Prevalence of infections and co-infections of HBV, HIV, and *Cryptosporidium*

	No. of infected participants	Infection rate
HIV	14	2.3
HBV	51	8.3
*Cryptosporidium*	74	12.0
Co-infection		
HIV and HBV	2	0.3
HIV and *Cryptosporidium*	2	0.3
HBV and *Cryptosporidium*	11	1.8

**Table 3:** Crude association between HBV infection and *Cryptosporidium* infection

	HBV infection [*n* (%)^a^]			*χ* ^*2*^	*P*
*Cryptosporidium* infection	Yes	No	Total		
Yes	11 (15.1)	62 (84.9)	73	5.00	0.03
No	40 (7.4)	502 (92.6)	542		

^a^Percentage in group

The results of the univariable and multivariable analyses for risk factors associated with *Cryptosporidium* infection are summarized in Table 4. The significant risk factors for acquiring *Cryptosporidium* infection were village of residence, keeping livestock or poultry, and being infected with HBV. After adjusting for covariates, an *OR* of 2.27 (95% *CI*: 1.01–5.08, *P* < 0.05) was associated with keeping livestock or poultry, and 3.42 (95% *CI*: 1.47–7.92, *P* < 0.01) with HBV infection. HIV infection was not found to be significantly associated with *Cryptosporidium* infection (a*OR* = 0.57, 95% *CI*: 0.07–4.39, *P* = 0.59). No significant associations were observed between *Cryptosporidium* infection any hygiene habits including drinking unboiled water, eating raw food, washing hands before meals or after defecation, and owning a household lavatory.

**Table 4:** Univariable and multivariable analyses for risk factors associated with *Cryptosporidium* infection

Variables	No. of participants	Infection rate (%)	c*OR* (95% CI)	*P*	a*OR* (95% CI)	*P*
Village						
A	172	16.3	1.00	0.02	1.00	0.02
B	158	14.6	0.88 (0.48–1.60)	0.67	0.81 (0.39–1.70)	0.58
C	129	10.1	0.58 (0.29–1.16)	0.12	0.47 (0.21–1.08)	0.08
D	156	5.8	0.32 (0.14–0.69)	<0.01	0.26 (0.10–0.64)	<0.01
Gender						
Male	251	13.5	1.00			
Female	364	10.7	0.77 (0.47–1.25)	0.29		
Age (years)						
0–10	110	12.7	1.00	0.24		
11–20	75	6.7	0.49 (0.17–1.42)	0.19		
21–30	87	10.3	0.79 (0.33–1.93)	0.61		
31–40	118	8.5	0.64 (0.27–1.50)	0.30		
41–50	110	17.3	1.43 (0.68–3.02)	0.35		
51–60	64	10.9	0.84 (0.32–2.21)	0.73		
>60	51	17.6	1.47 (0.59–3.66)	0.41		
Marital status						
Not married	194	11.3	1.00	0.74		
Married	383	11.7	1.04 (0.61–1.79)	0.89		
Widowed	38	15.8	1.47 (0.55–3.90)	0.44		
Body mass index (BMI, kg/m^2^)						
>18	526	12.2	1.00			
≤18	89	10.1	0.81 (0.39–1.70)	0.58		
Education level						
No school	382	12.0	1.00	0.85		
Primary school	206	11.2	0.92 (0.54–1.56)	0.75		
Middle school and above	27	14.8	1.27 (0.42–3.84)	0.67		
Occupation						
Student	113	9.7	1.00	0.72		
Farmer	492	12.4	0.76 (0.39–1.50)	0.43		
Other	10	10.0	0.79 (0.10–6.31)	0.82		
Annual family income(yuan)						
<3 000	117	10.3	1.00	0.63		
3 000–5 000	188	12.8	1.28 (0.61–2.67)	0.51		
5 000–10 000	116	14.7	1.50 (0.68–3.31)	0.31		
>10 000	194	10.3	1.01 (0.47–2.14)	1.00		
Keeping livestock or poultry (e.g., cattle, sheep, chicken)						
No	155	7.1	1.00		1.00	
Yes	460	13.5	2.04 (1.05–3.98)	0.04	2.27 (1.01–5.08)	<0.05
Drinking unboiled water						
No	14	14.3	1.00			
Yes	589	12.1	0.82 (0.18–3.75)	0.80		
Washing hands before meals						
Often or always	352	12.2	1.00			
Never or occasionally	253	11.9	0.97 (0.59–1.59)	0.89		
Washing hands before defecation						
Often or always	329	12.5	1.00			
Never or occasionally	277	11.6	0.92 (0.56–1.50)	0.73		
Washing fruits and raw vegetables before eating them						
Often or always	183	13.3	1.00			
Never or occasionally	392	10.4	0.76 (0.43–1.32)	0.33		
Having a household lavatory						
No	239	12.6	1.00			
Yes	352	11.9	0.94 (0.57–1.56)	0.82		
Living under the same roof with livestock						
No	541	12.6	1.00			
Yes	35	8.6	0.65 (0.19–2.19)	0.50		
HIV infection						
No	601	12.0	1.000			
Yes	14	7.1	0.57 (0.07–4.39)	0.59		
HBV infection						
No	564	11.0	1.00		1.00	
Yes	51	21.6	2.23 (1.09–4.56)	0.03	3.42 (1.47–7.92)	<0.01

## Discussion

The study area had a very high prevalence of *Cryptosporidium* infection (12.0%), according to modified acid-fast staining. The prevalence of *Cryptosporidium* infection was much higher than the previously estimated range of 0.79–10.40% among either individuals without diarrhea or patients with diarrhea in other parts of China [7, 9, 13]. In particular, our data showed that the prevalence was highest among participants aged 41–50 years, followed by children aged 0–10 years and elderly adults aged ≥ 60 years, which was similar to results of other studies [14, 15]. The high burden of *Cryptosporidium* infection may be attributed to poor personal hygiene and lack of sanitation facilities. The participants were predominantly Yi people with similar occupations, cultural traditions, and living conditions. For instance, only 2.3% of the participants would boil water before drinking it and nearly half of them had no access to a household lavatory. Not using sanitation facilities and water treatment and poor hygiene habits of some of the participants might increase the general environmental contamination and thereby increases the risk of *Cryptosporidium* infection for all other persons living in the same setting [16]. Therefore, the protective effects of sanitation and hygiene habits could have been underestimated. Moreover, owing to its low latitude and high elevation, the town studied has a mild climate and belongs to the humid subtropical zone. A recent meta-analysis examining the effects of seasonality showed that increases in both temperature and rainfall were associated with an increased risk of cryptosporidiosis [17]. These local climatic conditions define a typical habitat area for *Cryptosporidium* species. Alternatively, these differences may reflect the use of different diagnostic techniques. In addition, we found that participants who raised livestock or poultry were more likely to be infected with *Cryptosporidium*, which was consistent with previous studies conducted elsewhere [18–21].

The prevalence of HBV infection observed in our study corroborated previous nationwide large-scale epidemiological studies [22]. The majority of individuals found to be HBV-positive had not received appropriate treatment for HBV infection given they were diagnosed for the first time. Therefore, screening and diagnosis of HBV are of great importance for reducing the risk of HBV transmission and subsequent HBV-related diseases. Intriguingly, we found that HBV infection and *Cryptosporidium* infection were significantly associated with each other. For chronic hepatitis B, long-lasting virus persistence is related to a lack of memory T-cell maturation and severely impaired T-cell function, followed by a suppressed production of primary cytokines [23–25], which may increase the susceptibility to *Cryptosporidium* infection. A recent study demonstrated that the infection rate of *Cryptosporidium spp.* was higher in chronic hepatitis B patients than in healthy controls [26]. On the other hand, *Cryptosporidium* infection leads to an imbalance between Th1 and Th2 cytokines, along with inflammatory damage to the host’s small intestine [3, 27], which may facilitate the occurrence of HBV infection. Further research is required to explore the host immune responses to co-infection with HBV and *Cryptosporidium*. Such investigations would help interpret this finding and explore the clinical significance of cryptosporidiosis in patients infected with HBV.

The prevalence of HIV infection was 2.3%, which was much higher than the provincial and national levels, 0.170% and 0.058%, respectively [28, 29]. The vast majority of the participants were Yi people, who live in one of the poorest areas in China. Previous studies have shown that poverty is associated with HIV infection [30, 31]. Additionally, the local custom requests a man to marry his brother’s widow in order to maintain the family lineage, even if his brother have died of HIV-related illnesses or his sister-in-law has been diagnosed as HIV infection. Casual sex without condom also facilitates the spreading of HIV [32]. The prevalence of *Cryptosporidium* infection was 7.1% (1/14) in patients infected with HIV, which was in agreement with observations elsewhere [8, 33]. There was no significant association found between HIV infection and *Cryptosporidium* infection in our study. An earlier survey found a higher prevalence of *Cryptosporidium* among AIDS patients who refused antiretroviral therapy (ART) (21.21%; 7/33) than those who accepted regular treatment (4.25%; 9/212) [8]. The number of people with HIV infection was small in this study. Another reason may be that these HIV-positive individuals were not immunocompromised yet because the majority of the HIV-infected individuals were diagnosed for the first time and were at the early stage of the disease. As a result, they received no ART or were rarely treated. Along with the development of HIV infection and weakening of immunity, cryptosporidiosis in patients with HIV infection could be a major public health issue given the lack of an effective specific therapy or vaccine. Therefore, local governments and CDCs need to make more efforts in expanding access to and coverage of ART for patients with HIV infection.

There are some limitations to this study. Firstly, only one stool sample was collected from each participant. Given the intermittent shedding pattern of *Cryptosporidium* oocysts, the examination based on a single sample may lead to an underestimated prevalence of *Cryptosporidium* infection. Secondly, males were underrepresented because many young males in rural areas drift to the cities for better work opportunities. Additionally, our study used modified acid-fast staining to detect the existence of *Cryptosporidium* oocysts. However, misclassification may occur because the sensitivity and specificity of this method are not high [1]. To minimize the risk of misclassification, each smear was read by two separate technicians without knowing the study participant’s identity. One-tenth of the examined slides were randomly selected and re-examined for quality control. Moreover, it should be noted that the modified acid-fast staining employed in our study does not discriminate between *Cryptosporidium* species and subtypes, without which it is impossible to determine the infectious source of *Cryptosporidium* in this setting. Genotyping and subtyping tools have been used increasingly in the characterization of the transmission of *Cryptosporidium* [34–36]. Further research could include molecular analysis in order to determine the roles of anthroponotic and zoonotic transmission of *Cryptosporidium* in rural China. In view of these limitations, the findings of this study should be interpreted with caution when being generalized to the larger population and compared with results from other studies.

## Conclusions

In conclusion, there was a high prevalence of *Cryptosporidium* infection among residents in the community under investigation. An association was found between HBV infection and *Cryptosporidium* infection. Since no effective specific therapeutic agent for cryptosporidiosis is available at present, fenced husbandry, health education, and environmental sanitation programs might help reduce the occurrence of *Cryptosporidium* infections, and so could hepatitis B vaccination as well as timely ART for patients with HIV. Further research is required to determine the clinical significance of this parasite in individuals infected with HBV, and the interaction between HBV and *Cryptosporidium*.

## Additional file

Additional file 1: Multilingual abstracts in the five official working languages of the United Nations. (PDF 702 kb)

## Abbreviations

AIDS: Acquired immune deficiency syndrome
a*OR*: Adjusted odds ratio
ART: Antiretroviral therapy
CDC: Center for Disease Control and Prevention
*CI*: Confidence interval
c*OR*: Crude odds ratio
HBV: Hepatitis B virus
HIV: Human immunodeficiency virus
*OR*: Odds ratio
