# Supplementary material for: Prevalence of and risk factors associated with Cryptosporidium infection in an underdeveloped rural community of southwest China
Source: Infect Dis Poverty. 2017 Jan 9;6:2. doi: 10.1186/s40249-016-0223-9 (PMC5267368; doi:10.1186/s40249-016-0223-9)
Supplement: Additional file 1: — Multilingual abstracts in the five official working languages of the United Nations. (PDF 702 kb) [file 40249_2016_223_MOESM1_ESM.pdf]

Translation of the abstract into the five official working languages of the United Nations

تراجع انتشار البلهارسيا المونسونية في عُمان

ادريس العبيداني، ومحمد شعبان، وساتيش غوجي، وسالم الكثيري، وخالد المشيخي، وأمادو غربا، وألبيس فرانسيسكو غابريلي

ملخّص:

معلومات أساسية: أُكتشفت أول حالة إصابة بداء البلهارسيا المعوية، الذي تسببه البلهارسيا المونسونية، للمرة الأولى في عُمان عام 1979م. ويشير الاتجاه إلى إصابة الانسان بالبلهارسيا الطفيلية، والبلهارسيا السيريلوجية في المنطقة الموبوءة كان خلال الفترة من 1982-2014م، وتطابق البيانات التي أعدها الجهاز الوطني للرصد والتقييم، ومعايير القضاء على البلهارسيا، التي حددتها وزارة الصحة العمانية.

الطُرُق: أُجريت اختبارات على الطفيل والمصل على السكان (ولا سيما الأطفال) الذين يعيشون في المنطقة المعرضة لخطر البلهارسيا، في ظفار، وهي المنطقة الوحيدة الموبوءة في البلاد، على مدى ثلاثين عاماً. واستخدمت في الفحوصات طريقتا كاتو-كاتز المكثفة، ومقايسة التراص الدموي غير المباشر.

النتائج: تشير البيانات إلى انخفاض تدريجي لمعدل الإصابة بمرض البلهارسيا المنسونية خلال حقبتي الثمانينيات والتسعينيات، والظهور مرة أخرى في أوائل عام 2000م، وانخفاضاً كبيراً ملحوظاً بعد تنفيذ 6 جولات من العلاج الجماعي بعقار البرازيكوانتيل في الفترة من 2007 وحتى 2013م. وكان آخر حالات الإصابة بالطفيل 0٪ (2011)، بينما كانت آخر إصابة بالسيريولوجي (2014) 0,11٪.

الخلاصة: تراجعت معدلات الإصابة بالبلهارسيا في عمان مستويات متدنية. وتحقق معيار القضاء على المرض الذي وضعته وزارة الصحة العمانية (معدل الإصابة الطفيلية ≤ 1٪، والإصابة السيرولوجية ≤ 5٪) منذ عام 2008م. وهناك حاجة لمزيد من التقصي لتحديد ما إذا كان الحد من الانتشار قد تحقق في بعض البؤر، أو جميعها، وذلك في ضوء وضع إجراء تحقق رسمي بإشراف منظمة الصحة العالمية.

Translated from English version into Arabic by Hassan Adam, through

TRANSLATORS
WITHOUT BORDERS

我国西南农村地区隐孢子虫感染及影响因素分析

杨亚，周艺彪，肖鹏磊，史妍，陈跃，梁松，依火伍力，宋秀霞，姜庆五

摘要：

背景：隐孢子虫是一种人畜共患寄生性原虫，可引起人、家畜及野生动物的腹泻。作为一个重要的公共卫生问题，隐孢子虫在中国农村地区的流行现状尚不明确。本研究目的在于了解中国西南农村地区隐孢子虫感染状况，并探讨其危险因素。

方法：选择中国西南某彝族聚居区域的一个乡作为调查地点，于 2014 年对 687 位村民进行 HIV 和 HBV 筛查，同时采用改良抗酸染色法检测隐孢子虫卵囊。采用单因素分析和多因素 Logistic 回归模型对感染的影响因素进行分析。

结果：共调查 687 人，其中绝大多数为彝族，多没有良好的卫生习惯。纳入分析的 615 人中，HIV 阳性 14 人，阳性率为 2.3%；HBV 阳性 51 人，阳性率为 8.3%；隐孢子虫粪检阳性 74 人，阳性率为 12.03%。HIV/HBV，HIV/隐孢子虫和 HBV/隐孢子虫合并感染率分别为 0.3%，

0.3%和 1.8%。隐孢子虫感染者中 HBV 感染率较高，差异有统计学意义（χ^2=5.00，p=0.03）。多因素分析表明，饲养家畜或家禽（aOR= 2.27，95%CI：1.01，5.08， $P < 0.05$）和 HBV 感染（aOR = 3.42， 95%CI：1.47，7.92，$P < 0.01$）与隐孢子虫感染有关，而 HIV 感染与隐孢子虫感染无关（aOR=0.57，95%CI：0.07，4.39， $P = 0.59$）。
结论：中国西南农村地区隐孢子虫感染率较高。HBV 感染与隐孢子虫感染显著相关，但是两者之间的关系仍然需要进一步研究。

Translated from English version into Chinese by Ya Yang

Diminution du taux de transmission de la schistosomiase due au Schistosomia mansoni à Oman

Idris Al Abaidani, Seif Al-Abri, Mahmoud Shaban, Satish L Ghugey, Salem Al Kathery, Khalid Al-Mashikhi, Amadou Garba, Albis Francesco Gabrielli

Résumé

Contexte : la schistosomiase intestinale due au Schistosomia mansoni a été signalée pour la première fois à Oman en 1979. Nous nous attachons à décrire la tendance en termes de prévalence parasitologique et sérologique de l'infection humaine par le S. mansoni dans sa région endémique de 1982 à 2014 et la conformité des données générées par le système national de suivi et d'évaluation par rapport aux critères d'élimination de la schistosomiase définis par le Ministère de la Santé d'Oman.
Méthodes : les évaluations parasitologiques et sérologiques ont été réalisées sur une population (principalement infantile) vivant dans la région à risque de contraction de la schistosomiase à Dhofar, le seul gouvernorat endémique du pays, pendant une période de plus de 30 ans. La mesure sur goutte épaisse de Kato-Katz et le test d'hémagglutination indirecte ont à cette occasion été utilisés.
Résultats : les données indiquent une diminution progressive de la prévalence du S. mansoni tout au long des années 1980 et 1990, une recrudescence au début des années 2000, puis une baisse plus marquée à la suite de la mise en œuvre de six séries de traitement de masse au praziquantel de 2007 à 2013. Le dernier taux de prévalence parasitologique (2011) atteignait 0 %, tandis que le dernier taux de prévalence sérologique (2014) s'élevait à 0,11 %.
Conclusions : la transmission de la schistosomiase a atteint des taux très faibles à Oman. Les critères d'élimination établis par le Ministère de la Santé d'Oman (prévalence parasitologique < 1 % et prévalence sérologique < 5 %) sont satisfaits depuis 2008. De plus amples examens sont requis afin d'évaluer si l'interruption de la transmission a été obtenue dans certains ou tous les foyers afin d'établir un processus de vérification formelle sous l'égide de l'OMS.

Translated from English version into French by eric ragu, through

TRANSLATORS
WITHOUT BORDERS

Воздействие врожденных и адаптационных иммунных ответов на дифференциальный клинический исход лепры

Адриана Барбоса-де-Лима-Фонсека, Марис До-Вале-Симон, Родриго Ансельмо-Казханига, Татьяна Родригез-де-Маура, Роке Рачеко-де-Альмейда, Малькольм С. Дати, Стивен Дж. Рид, Амелия Рибейро-де-Хесус

Краткое изложение

Лепра – это хроническое инфекционное заболевание, вызываемое микобактериями *Mycobacterium leprae*. По данным официальных исследований в 121 стране по пяти регионам, охватываемым Всемирной организацией здравоохранения, в 2014 году было поставлено 213 899 новых диагнозов лепры. Хотя лепра поражает кожу и периферические нервы, она может проявляться в самых различных клинических и гистопатологических формах, которые сильно связаны с иммунной реакцией пациента. Эти формы включают в себя такие экстремальные случаи, как туберкулоидная лепра (ТТ), с *M. leprae*-специфическим Th1, а также Th17, иммунным ответом, ограничивающим размножение *M. leprae*, а также лепроматозную лепру (LL), с *M. leprae*-специфическими Th2 и T ответами, не контролирующими репликацию *M. leprae*,, а позволяющими распространение бактерий. Биполярные пограничные клинические формы демонстрируют похожие, но менее экстремальные иммунные ошибки. Периоды острого воспаления, называемые лепрозными реакциями, являются осложнениями, которые могут происходить до лечения, во время или после него, и приводят к дальнейшим неврологическим повреждениям, которые могут вызвать необратимую хроническую инвалидность. Данный обзор рассматривает врожденные и адаптационные иммунные ответы и их взаимодействие, влияющие на патогенез и клинический исход заболевания.

Translated from English version into Russian by Elena McDonnell, through

TRANSLATORS
WITHOUT BORDERS

Disminución de la transmisión de esquistosomiasis mansoni en Omán

Idris Al Abaidani, Seif Al-Abri, Mahmoud Shaban, Satish L Ghugey, Salem Al Kathery, Khalid Al-Mashikhi, Amadou Garba, Albis Francesco Gabrielli

Resumen

Antecedentes: La esquistosomiasis intestinal provocada por *Schistosoma mansoni* fue observada por primera vez en Omán, en 1979. Describimos la tendencia de la prevalencia parasitológica y serológica de la infección en humanos con *S. mansoni* en su área endémica durante el periodo 1982-2014, y el cumplimiento de los datos generados por el sistema nacional de seguimiento y evaluación

con los criterios de eliminación de la esquistosomiasis establecidos por el Ministerio de Sanidad de Omán.

Métodos: Durante un periodo de 30 años, se realizaron evaluaciones parasitológicas y serológicas en la población (principalmente niños) que habita en la zona de riesgo para la esquistosomiasis de Dhofar, la única gobernación del país donde es endémica. Las técnicas empleadas fueron la técnica de frotis grueso de Kato-Katz y la hemaglutinación indirecta.

Resultados: Los datos muestran una reducción progresiva de la prevalencia de *S. mansoni* a lo largo de las décadas de los 80 y los 90, un agravamiento a comienzos de la década de 2000, y un descenso más acusado tras la aplicación de seis tandas de tratamiento masivo con praziquantel desde 2007 hasta 2013. La última prevalencia parasitológica (2011) fue del 0% mientras que la última prevalencia serológica (2014) fue del 0,11%.

Conclusiones: La transmisión de la esquistosomiasis ha llegado a niveles muy bajos en Omán. Desde 2008 se han cumplido los criterios de eliminación establecidos por el Ministerio de Sanidad de Omán (prevalencia parasitológica ≤ 1% y prevalencia serológica ≤ 5%). Es necesario realizar investigaciones adicionales para evaluar si la interrupción de la transmisión se ha logrado en todos los focos o solo en algunos, con vistas a establecer una proceso de verificación formal bajo los auspicios de la OMS.

Translated from English version into Spanish by Barbara Gutierrez Teira, through

TRANSLATORS
WITHOUT BORDERS
